# Bronchopulmonary Dysplasia: Crosstalk Between PPARγ, WNT/β-Catenin and TGF-β Pathways; The Potential Therapeutic Role of PPARγ Agonists

**DOI:** 10.3389/fped.2019.00176

**Published:** 2019-05-03

**Authors:** Yves Lecarpentier, Elizabeth Gourrier, Vincent Gobert, Alexandre Vallée

**Affiliations:** ^1^Centre de Recherche Clinique, Grand Hôpital de l'Est Francilien, Meaux, France; ^2^Service de néonatologie, Grand Hôpital de l'Est Francilien, Meaux, France; ^3^Diagnosis and Therapeutic Center, Hypertension and Cardiovascular Prevention Unit, Hôtel-Dieu Hospital, AP-HP Paris, Paris-Descartes University, Paris, France

**Keywords:** bronchopulmonary dysplasia, canonical WNT/β-catenin, TGF-β, PPARγ, fibrosis, myofibroblast, nebulized thiazolidinediones

## Abstract

Bronchopulmonary dysplasia (BPD) is a serious pulmonary disease which occurs in preterm infants. Mortality remains high due to a lack of effective treatment, despite significant progress in neonatal resuscitation. In BPD, a persistently high level of canonical WNT/β-catenin pathway activity at the canalicular stage disturbs the pulmonary maturation at the saccular and alveolar stages. The excessive thickness of the alveolar wall impairs the normal diffusion of oxygen and carbon dioxide, leading to hypoxia. Transforming growth factor (TGF-β) up-regulates canonical WNT signaling and inhibits the peroxysome proliferator activated receptor gamma (PPARγ). This profile is observed in BPD, especially in animal models. Following a premature birth, hypoxia activates the canonical WNT/TGF-β axis at the expense of PPARγ. This gives rise to the differentiation of fibroblasts into myofibroblasts, which can lead to pulmonary fibrosis that impairs the respiratory function after birth, during childhood and even adulthood. Potential therapeutic treatment could target the inhibition of the canonical WNT/TGF-β pathway and the stimulation of PPARγ activity, in particular by the administration of nebulized PPARγ agonists.

## Introduction

Bronchopulmonary dysplasia (BPD) is a particularly serious pulmonary disease occurring in preterm infants (1). In the USA, 1.6% of all births are premature (<32 weeks of gestation); 1.1% have a birth weight <1.5 kg, of which 25% will develop a og BPD (2). Thus, there are about 10,000–15,000 new cases every year. Current therapies are usually ineffective and, despite all necessary precautions being taken, some are even abandoned due to possible aggravation of the clinical pattern. In BPD, PPARγ has been shown to be downregulated in several animal models, while the canonical WNT/β-catenin pathway has been upregulated. In numerous pathologies, these two pathways are observed to function in an opposite manner (3, 4). PPARγ is upregulated in some diseases, while the WNT/β-catenin system is downregulated (5). However, the inverse is observed in many other diseases (6). The absence of a decrease in the WNT/β-catenin signaling during the canalicular stage of pulmonary development, partly related to inflammatory processes, a hallmark of BPD, will seriously affect normal development in the subsequent saccular and alveolar stages (7). This is responsible for the respiratory distress at birth and the often severe and irreversible sequelae observed in childhood and adulthood. Several animal studies have reported beneficial effects induced by nebulized PPARγ agonists but no studies to date have been performed on human neonates. In the present study, we review the respective roles in BPD of both the canonical WNT pathway and PPARγ, which are determined by TGF-β signaling, and discuss their possible implications for the therapeutic treatment of BPD.

### Overview of BPD

BPD was first described by Northway et al. (1). Most premature infants suffer from respiratory distress and surfactant deficiency with pulmonary lesions resulting from the association of multiple infections and inflammation, and possibly aggravated by mechanical ventilation and oxygen toxicity. In BPD, all lung tissues are impaired and airway inflammation, bronchiolitis, alveolar collapse coexist (8). Surviving premature infants often present an abnormal respiratory function when they become adolescents or adults (9, 10). Over the last 20 years, the survival of preterm infants with BPD has been improved thanks to perinatal care, including the use of surfactant and new strategies for mechanical ventilation and oxygenation, and the cure of ductus arteriosus (11). On the other hand, improved survival rates have resulted in an increased incidence of long-term BPD complications. BPD is characterized by an arrest of the alveolization stage. BDP is secondary to pulmonary aggressions occurring at a strategic moment of lung morphogenesis, particularly at the canalicular stage (7, 12–14). The basic histological description reports a decrease in alveolization and an activation of fibroblasts that differentiate into myofibroblasts (12, 15) which express α-smooth muscle actin (α-SMA) in alveolar septa (16). No therapy consistently enhances lung maturity postnatally (17–19). Myofibroblasts exhibit platelet-derived growth factor (PDGF) receptors and express α-SMA and elastin, which are necessary for alveologenesis (20–26). During tracheal aspiration in children who subsequently develop normal lung function, there are almost no mesenchymal stem cells (MSCs) in the suction fluid (27). In contrast, MSCs are found in the suction fluid of premature infants with respiratory distress. TGF-β induces myofibroblastic differentiation of MSCs. About half of the children in whom MSCs have been isolated will develop a BPD (28). α-SMA is higher in differentiated MSCs in children developing BPD than in those who do not develop it. This reflects a greater amount of differentiated myofibroblasts. The transdifferentiation of pulmonary lipofibroblasts into myofibroblasts also represents an important element in the pathogenesis of BPD (23, 29).

### Normal Lung Organogenesis in Humans

**Figure 1:**
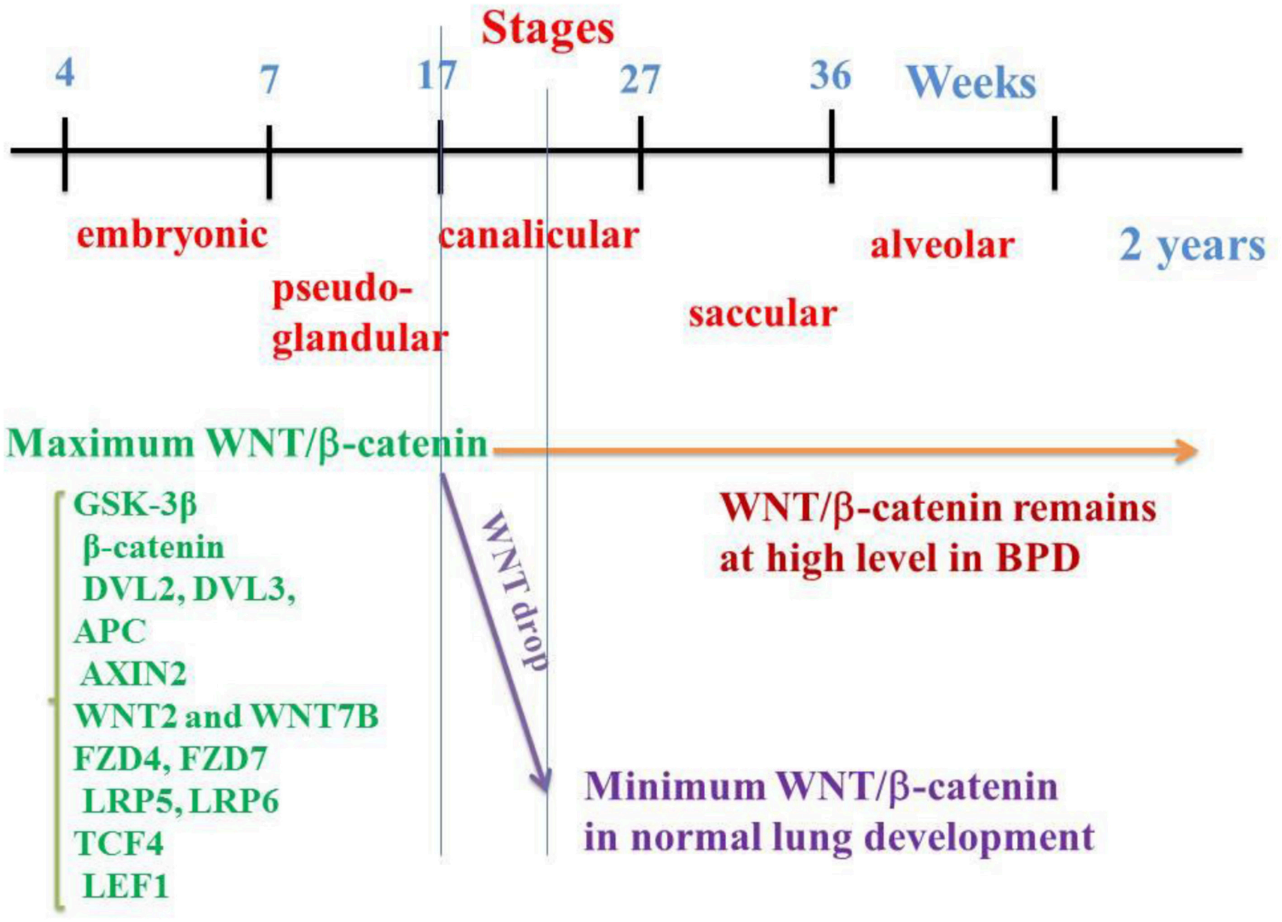
WNT/β-catenin pathway on the embryological pulmonary development in humans. Five stages classically follow one another: embryonic, pseudo-glandular, canalicular, saccular, and alveolar. WNT/β-catenin signaling reaches a maximum activation at the 17th week (end of the pseudo glandular stage or beginning of the canalicular stage). In the middle of the canalicular stage (around the 21st week), canonical WNT pathway activity decreases dramatically, a necessary prerequisite for the correct realization of the alveolar stage. In the event of premature birth, the thick alveolar walls do not allow sufficient gas exchanges, which can lead to pulmonary hypoxia. Hypoxia activates the canonical WNT/β-catenin, impairs the alveolar stage, and promotes the synthesis of myofibroblasts and subsequent fibrosis, with abnormalities in the respiratory function.

Several cascades are implied in lung organogenesis, such as bone-morphogenic proteins (BMPs), fibroblast growth factors (FGFs), sonic hedgehog (SHH), and the WNT family (30–33). Human lung morphogenesis begins after ~4 weeks of gestation and continues into postnatal life up to early adulthood. At around 4 weeks of gestation, the lung buds originate as an outgrowth from the foregut ventral wall where the lobar division appears. In humans, lung development is traditionally divided into five stages based on changes in the airway tubular structure and epithelial cells as follows (34) (Figure 1):

1. ° Embryonic stage (4–7 weeks *in utero*): lung buds are formed and the trachea begins branching in lobar and segmental bronchi.
2. ° Pseudoglandular stage (7–17 weeks): at about 7 weeks, the airways branch and epithelial cells differentiate into tall pseudostratified epithelium. At ~12 weeks, small tubular structures are surrounded by short columnar cells. About 70% of the total airways are formed at 14 weeks.
3. #x000B0; Canalicular stage (17–27 weeks): cuboidal cells are progressively replaced by a columnar epithelium. All of the conducting airways and terminal bronchioles are formed at 18 weeks. At 21 weeks, the differentiation of pneumocytes appears.
4. ° Saccular stage (27–36 weeks). At this stage, alveolar ducts and air sacs are present. The number of saccules increases and secondary ridges begin to form with extensive vasculogenesis at the level of the terminal saccules.
5. ° Alveolar stage (36 weeks of gestation to 2 years of age): the secondary septation occurs with a marked increase in number and size of alveoli and capillaries. There is a rapid increase in lung volume and alveolar area (35). At 36 weeks, all cells are uniformly present, and their number increases exponentially with the gestational age. Lipofibroblasts leading to type II interstitial cells are present at the septa bases and regulate the lipid metabolism for the surfactant phospholipid synthesis.

### Hypoxia Impairs Lung Development in BPD

Hypoxic episodes occur in BPD preterm infants partly due to immature respiratory control. Relatively short respiratory pauses may precipitate O2 desaturation, bradycardia, and pulmonary hypertension. This can induce retinopathy of prematurity (ROP), sleep disordered breathing, and neurodevelopmental delay (36, 37).

The organogenesis of the respiratory system in premature infants has an adverse effect on pulmonary ventilation and gas exchange. Indeed, the surfactant deficiency maintains the pulmonary compliance at a low level and the alveolar wall thickness limits the diffusion of oxygen and carbon monoxide. For premature infants, an additional supply of oxygen and mechanical ventilation is proposed at this stage, especially as the compliance of the chest wall is low, helping to lower the functional residual capacity and to increase the likelihood of respiratory failure. With such prematurity, the alveolar stage cannot be carried out correctly. Once born, the premature infant must breathe independently. Thickened alveolar walls and vascular pulmonary abnormalities impair effective gas diffusion leading to lung tissue hypoxia, which impairs lung development (38, 39). Importantly, hypoxia is probably the *primum movens* which activates the canonical WNT pathway and inactivates PPARγ (40). During hypoxia, the respiratory chain increases the ROS production leading to increase hypoxia-induced factor (HIF)-dependent gene expression which represents an important regulator of glycolysis energy metabolism involved in the pathogenesis of lung fibrosis (41).

Conversely, the oxygen therapy that is sometimes necessary can induce a hyperoxia which also activates the canonical WNT signaling and inactivates PPARγ.

### Lung Lesions Due to Hyperoxia and Volutrauma Mechanical Ventilation

The role of hyperoxia in BPD is now well-established (42, 43). In animals, hyperoxia alone can stop the pulmonary septation at the saccular stage of lung development (44, 45). Neonatal resuscitation of premature infants between 24 and 26 weeks (canalicular stage) with 30% O2 instead of 90% O2 decreases the incidence of BDP at 36 weeks by a factor of about 2 (46). In a newborn rat model of BPD induced by intra-amniotic LPS administration and postnatal hyperoxia, there is an arrest in alveolar and pulmonary vascular development, which is a hallmark of BPD (47–50). Hyperoxia-induced neonatal lung injury is associated with activation of Wnt/β-catenin signaling and inhibition of WNT/β-catenin signaling attenuates hyperoxia-induced pulmonary hypertension in neonatal rats (51, 52). Hyperoxia-induced neonatal lung injury is mediated via TGF-β activation (53, 54). Thus, p-Smad3 and 7, and TGF-β R2 receptor protein levels increase after hyperoxia exposure. Hyperoxia leads to pro-inflammatory factors such as TNF-α and IL-8 and reactive oxygen species (ROSs). Likewise, high tidal-volume mechanical ventilation increases the appearance of several pro-inflammatory agents (IL-1b, IL-6, IL-8) (55). This has led to the use of anti-oxidant agents in BPD as a preventive therapy (56, 57). Lungs exposed to hyperoxia show an increase in interstitial myofibroblasts that produce α-SMA and TGF-β (58–61). In the neonatal mouse, hyperoxia induces the expression of periostin in the alveolar wall and particularly in thickened interstitial areas (62). This is also observed on histological sections of infants who died from BPD. Moreover, periostin knockout mice are protected from alveolar complications generated by hyperoxia and do not show interstitial myofibroblasts. Hyperoxia induces an increase in Connective Tissue Growth Factor (CTGF or CCN2) mRNA and CTGF protein in lung epithelial cells and thickening of the alveolar wall (63). In animals, mechanical ventilation induces a pulmonary phenotype close to BPD (64–66). Paracrine effects between lung fibroblasts and epithelial cells are impaired after exposure to hyperoxia. This increases the likelihood of lipofibroblast transdifferentiation into myofibroblasts (54). Thus, both hyperoxia and hypoxia lead to impaired alveolization, myofibroblast differentiation, irreversible pulmonary fibrosis, and major alterations in lung function.

#### Oxygen Toxicity

Oxygen toxicity is in great part due to reactive oxygen species (ROSs) generated by mitochondrial respiratory chain, inflammation, hypoxia, ischemia, and hyperoxia (67). Antioxidant capacity is decreased in preterm newborns with deficiency of antioxidant factors (68, 69). ROSs following hyperoxia are responsible for injuries in lungs, central nervous system, retina, and red blood. Severe ROP is due to susceptibility of the phospholipid-rich retina to ROSs (70). To research the safest level of O2 saturation in preterm newborns requiring supplemental oxygenation is important to reduce the incidence of ROP. For some authors, the setting up oxygen alarms are below 85% of oxygen saturation and above 93% in newborns <32 weeks of gestation (71). Other authors have reported a decrease of incidence of ROP in newborns treated with lower O2 saturation (70–90%. rather 88–98%) and cognitive outcomes after 10 years in newborns treated with lower O2 saturation level (72). Several randomized controlled trials have studied the question of what are the optimal O2 saturation levels to reduce outcomes and especially incidence of ROP, chronic lung diseases and hospitalization duration (73, 74). In other randomized controlled trials, the same ranges of O2 saturation has been used in two different groups, i.e., 85–89% in the lower group vs. 91–95% in the higher group (75–77). Based on these randomized controlled trials, mechanical ventilation may be lifesaving but can cause lung injury. Supplemental oxygenation protocols must avoid mechanical ventilation when it is possible (78). In preterm newborns requiring supplemental oxygenation, the range of optimal O2 saturation remains elusive, due numerous different clinical conditions and gestational ages. It is important to avoid both hypoxia and hyperoxia and is recommended to maintain an intended optimal O2 saturation of 90–95% (70).

## Brief Overview of the Canonical WNT/β-catenin-TGF-β Pathway and PPARγ

### Canonical WNT/β-catenin Pathway (Figure 2)

**Figure 2:**
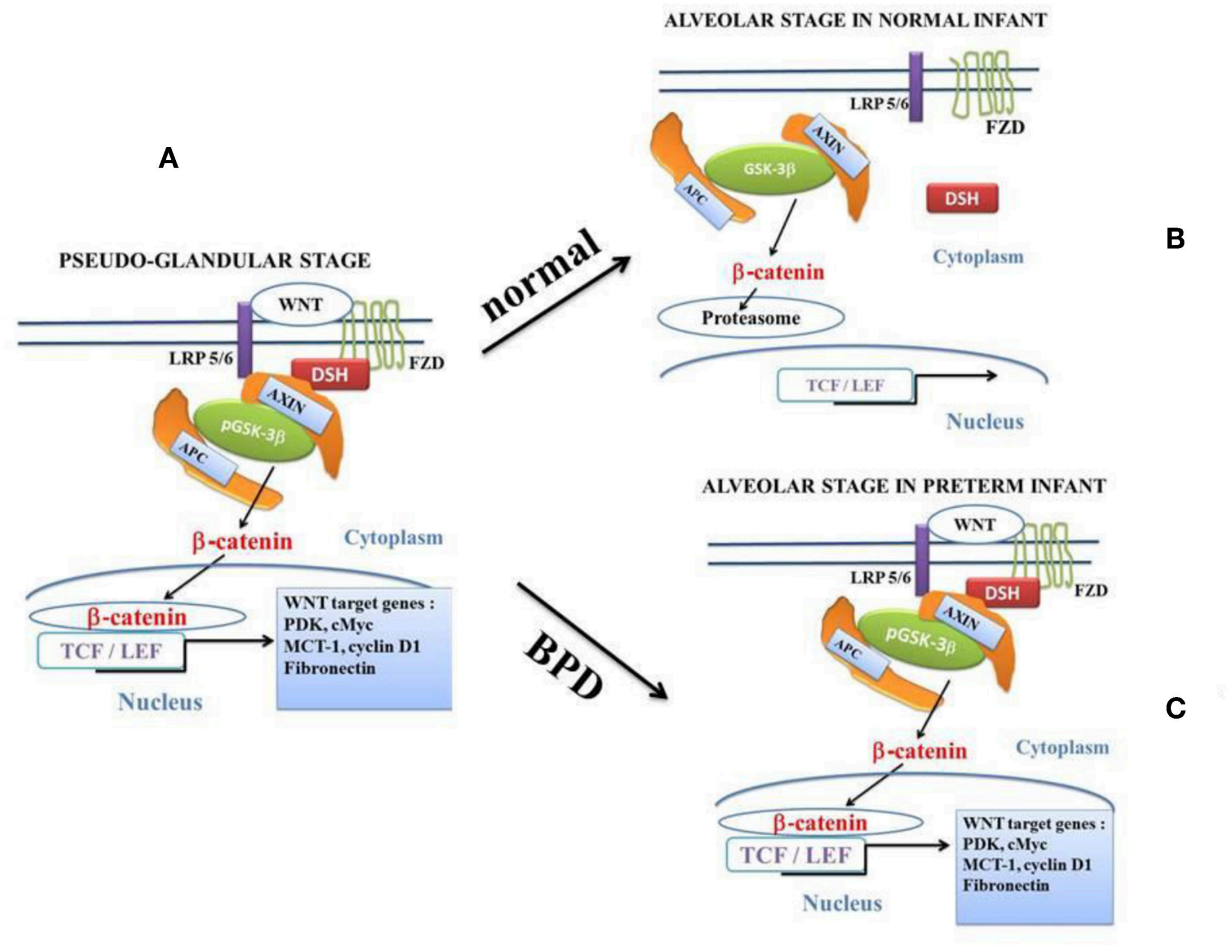
The canonical β-catenin/WNT pathway: “on” and “off” states. The hallmark of the canonical β-catenin/WNT pathway activation is the elevation of the cytoplasmic β-catenin protein level, the subsequent nuclear translocation of β-catenin and further activation of β-catenin specific gene transcription. The canonical β-catenin/WNT pathway can be either in “on-state” or in “off-state.” The pathway is in “on-state” in the presence of a WNT ligand that binds both Frizzled (FZD) and LRP5/6receptors. This leads to activation of the phosphoprotein Disheveled (DSH). DSH recruits the destruction complex (pGSK-3β + AXIN + APC) to the plasma membrane, where AXIN directly binds the cytoplasmic tail of LRP5/6. APC is the adenomatous polyposis coli and GSK-3β is the glycogen synthase kinase-3β. In “on-state,” pGSK-3β is inactivated which corresponds to the phosphorylated state (pGSK-3β). Activation of DSH leads to the inhibition of both phosphorylation and degradation of beta-catenin. Beta-catenin accumulates into the cytosol and then translocates to the nucleus to bind lymphoid-enhancing/T cell (LEF-TCF) co-transcription factors. This induces the WNT-response gene transcription. In the “off state,” in the absence of WNT ligand or in the presence of the active form of GSK-3β (i.e., the unphosphorylated form of GSK-3β), cytosolic β-catenin is phosphorylated by the active form of GSK-3β. Beta-catenin undergoes the destruction process into the proteasome. **(A)**: at the pseudo-glandular stage of the pulmonary development, the canonical WNT/β-catenin pathway is in “on-state.” **(B)**: at the saccular and alveolar states, and in normal infants, the canonical WNT/β-catenin pathway is in “off-state.” The pulmonary development is normal. **(C)**: at the saccular and alveolar states, in preterm infants with BPD, the canonical WNT/β-catenin pathway is in “on-state” and the pulmonary development is dramatically impaired.

**Figure 3:**
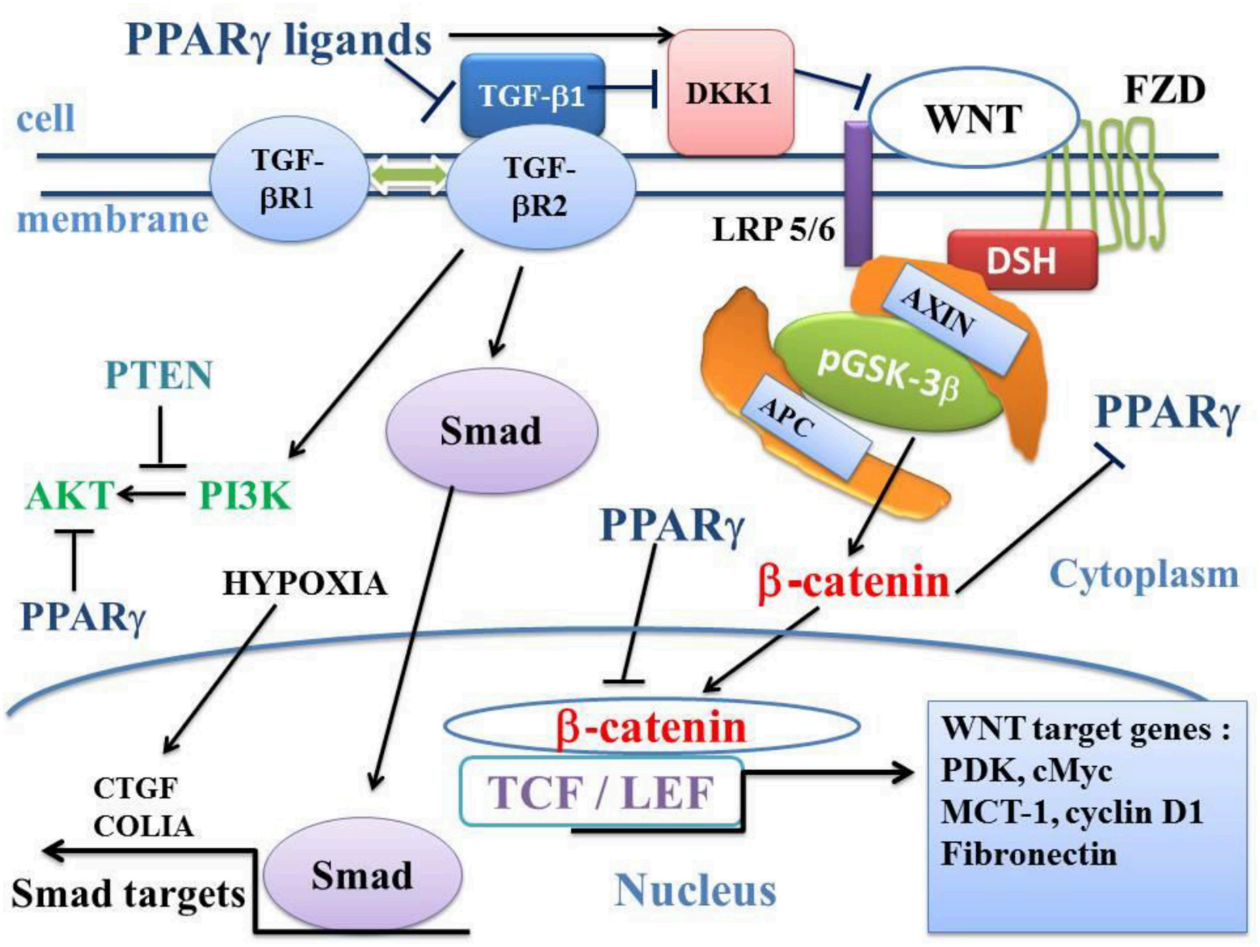
Influence of TGF-β1 on the balance between the canonical WNT/β-catenin signaling and PPARγ. In the presence of the WNT ligands, the WNT receptor binds both LRP5/6 and FZD receptors to initiate LRP phosphorylation and DSH-mediated Frizzled internalization. This leads to the dissociation of the GSK-3 β/AXIN/APC destruction complex. Phosphorylation of β-catenin is inhibited and β-catenin accumulates in the cytosol and then translocates to the nucleus to bind TCF-LEF transcription factors. This leads to the WNT-response gene transcription (PDK, MCT-1, cMyc, and Cyclin D1). PPARγ inhibits the β-catenin/TCF-LEF-induced activation of WNT target genes. TGF-β also enhances WNT signaling through the inhibition of DKK1. DKK1 is activated by PPARγ. TGF-β1 binds type 2 TGF-βR2 receptor (TGF-βR2), which recruits type 1 TGF-βR1 receptor (TGF-βR1). This results in the formation of a heterotetramer that phosphorylates Smad. The Smad complex then translocates to the nucleus and regulates the transcription of target genes (CTGF, COL1A). A non-Smad pathway also occurs through PI3K-AKT. PTEN inhibits PI3K-AKT and PPARγ inhibits AKT. APC, adenomatous polyposis coli; CTGF, Connective tissue growth factor; DKK1, Dickkopf-1; DSH, Disheveled; FZD, Frizzled; GSK-3β, glycogen synthase kinase-3β; LRP5/6, low-density lipoprotein receptor-related protein 5/6; MCT-1, monocarboxylate lactate transporter-1; PPARγ, peroxisome proliferator-activated receptor gamma; PI3K, phosphatidylinositol 3-kinase and AKT, AKT/Protein Kinase B; PTEN, Phosphatase and tensin homolog; PDK, pyruvate; dehydrogenase kinase; TCF/LEF, T-cell factor/lymphoid enhancer factor; TGF, Transforming Growth Factor.

The canonical WNT pathway plays a key role in embryogenesis, cell fate, metabolism and epithelial-mesenchymal transition (EMT) (79–81). In the presence of canonical WNT ligands (Figures 2, 3), the canonical WNT receptor is linked with Frizzled (FZD) and LDL receptor-related protein 5/6 (LRP5/6). FZD linked to Disheveled (DSH) disrupts the destruction complex [tumor suppressor adenomatous polyposis coli (APC), AXIN and glycogen synthase kinase-3β (GSK-3β)]. Then, β-catenin translocates to the nucleus and interacts with the T-cell /lymphoid enhancer (TCF/LEF) transcription factors to activate β-catenin target genes (82, 83). With no WNT ligands, β-catenin is phosphorylated by the destruction complex and degraded in the proteasome. β-catenin regulates the expression of numerous target genes such as cyclin D1, MMP7, c-Myc, fibronectin, etc., through interactions with TCF and LEF (84).

### Transforming Growth Factor (TGF-β)

TGF-βs are three proteins, TGF-β1, TGF-β2, and TGF-β3, and receptors are the transmembrane proteins Type I (TGFβRI) and Type II (TGFβRII). TGF-β1 activates the Smad pathway and non-Smad pathways such as MAPK, Rho, PI3K-AKT and downregulates PPARγ expression via the SMAD pathway (85) (Figure 3). In alveolar epithelial cells (AEC), TGF-β1 mediates epithelial-mesenchymal transition (EMT) and this leads to lung fibrosis. In idiopathic pulmonary fibrosis, myofibroblasts induce lung fibrosis (86–88). TGF-β1-induced EMT of AEC type II results in myofibroblast differentiation in fibrotic lungs (89–93).

### PPARγ

PPARγ is a ligand-dependent transcriptional factor belonging to the nuclear hormone receptor superfamily. PPARγ regulates glucose and lipid homeostasis, insulin sensitivity, inflammation, innate immune responses and cell fate (94, 95). PPARγ is activated by natural agents such as 15d- prostaglandin J2 (15d-PGJ2) and synthetic ligands including thiazolidinediones (TZDs). TZDs improve glucose tolerance and insulin sensitivity in type 2 diabetes. PPARγ dedifferentiates myofibroblasts, increases collagen uptake by lung alveolar macrophages, and may reverse fibrosis in an animal model (96). PPARγ plays an important role in normal lung development via epithelial-mesenchymal signaling (97).

### TGF-β1, WNT/β-catenin and PPARγ Interactions

There is a strong link between TGF-β1, canonical WNT/β-catenin and PPARγ (98, 99). TGF-β1 upregulates the WNT/β-catenin pathway, and inhibits PPARγ. TGF-β1 induces differentiation of human lung fibroblasts into myofibroblasts. Conversely, PPARγ inhibits the TGF-β1/WNT/β-catenin pathway. PPARγ ligands repress TGF-β1-induced myofibroblast differentiation via the PI3K/AKT signaling (100) (Figure 3). TGF-β1 induces fibrosis and represents a key therapeutic target in fibrotic processes (101, 102).

## Canonical WNT/β-catenin Pathway and Normal Lung Morphogenesis

In humans, the WNT/β-catenin pathway plays a key role in the patterning of early lung organogenesis, especially during the canalicular stage (7, 103–107). Regional specialization of the epithelium and mesenchyme and branching morphogenesis have been investigated by means of various techniques, such as reporter gene activity, misexpression, lineage tracing, β-catenin deletion/stabilization, WNT/ β-catenin in patterning of the mesenchyme and morphogenesis of proximal structures (108). During the pseudoglandular stage, most of the canonical WNT/β-catenin components appear to be already present in the lung buds. They are involved in the branching and division of conduction airways. The WNT pathway is not required for the lung primary branching pattern but is necessary for a correct branching morphogenesis (103). The canonical WNT/β-catenin components are mainly expressed in the alveolar and bronchial epithelium. An appropriate patterning of the developing lung is dependent on epithelial-mesenchymal cell interactions (32).

In humans, M. Zhang et al. (7) have determined the spatio-temporal patterns of the main components of the WNT/β-catenin signaling by means of qRT-PCR and *in situ* hybridization at 7, 12, 17, and 21 weeks of gestation. Among them are the GSK-3β, β-catenin, DVL2, DVL3, APC and AXIN2 components, WNT2 and WNT7B ligands, FZD4, FZD7, LRP5, and LRP6 receptors, and TCF4 and LEF1 transcription factors. Importantly, most of them are detected at 7 weeks, reach their maximum value at 17 weeks (onset of the canalicular stage) and dramatically decrease at 21 weeks (middle of the canalicular stage) (Figure 1). This is corroborated by other studies that show firstly, in the normal lung, the presence of β-catenin in the majority of epithelial and mesenchymal cell nuclei at 18 weeks of gestation with a decrease at the 21st week of gestation (14) and, secondly, a second-trimester β-catenin spike with attenuation of nuclear β-catenin at the alveolar stage of lung development (109). Other studies have shown a peak of the WNT signal in epithelial and mesenchymal cells toward the onset of the canalicular stage with a sharp decline toward the middle of the canalicular stage, and a low level during the saccular and alveolar stages (110). In human lung at 15 weeks (pseudo-glandular stage) and after exposure to CHIR 99021, a selective GSK-3β inhibitor (111), an increased expression of β-catenin transcription factors (TCF4, LEF1) and target genes (Cyclin D1, MMP7) have been observed. In human lung tissues *in vitro*, WNT3A induces an increase in mRNA levels of the WNT target gene Cyclin D1 (112). There is therefore a regulation of the WNT canonical system both temporally and spatially, which helps explain the pathophysiology of BPD.

## PPARγ and Physiological Lung Development

PPARγ has been shown to play a pivotal role in normal lung development and homeostasis by stimulating alveolar interstitial lipofibroblast maturation (113–115). Lipofibroblasts induce alveolar epithelial-mesenchymal paracrine interactions through the stimulation of surfactant phospholipid and by favoring endogenous antioxidants (113, 116, 117). PPARγ is critical for normal lung organogenesis and homeostasis of lung epithelium and mesenchyme. PPARγ is involved in normal lung development via EMT (97). PPARγ induces lipofibroblast differentiation which exerts a cytoprotective effect against oxidant injury (113, 116, 118). PPARγ stimulates trans-differentiation of myofibroblasts into lipofibroblasts, which helps normal alveolarization (119). PPARγ also favors the lipofibroblastic phenotype, which leads to the alveolar type II cell.

## The Canonical WNT/β-catenin Signaling in BPD (Figure 1)

BPD results from pulmonary airway aggregates during the saccular stage (27–36 W), after the peak of WNT/β-catenin activity which occurs in the normal lung at 17W, i.e., at the onset of the canalicular stage (7). In BPD, nuclear β-catenin in lung tissues is quantitatively close to that observed in week 17–18 of the normal lung (which corresponds to the peak of β-catenin in the normal fetus (7, 14). The WNT/β-catenin pathway remains in the “on-state” in the lung of premature infants in the saccular and alveolar stages, and in the postnatal period. Instead of dropping drastically at 21 weeks (i.e., in the middle of the canalicular stage), WNT/β-catenin signaling remains high, which leads to major alterations in pulmonary development, with significant abnormalities being observed at the alveolar stage. This makes the WNT/β-catenin pathway a potential therapeutic target for BPD (7, 14, 110, 120).

In BPD infants, the resting metabolic expenditure is elevated with growth failure and expression of some genes of oxidative phosphorylation is decreased (121). Overactivation of the Wnt/β-catenin pathway induces aerobic glycolysis where a part of the glucose supply is fermented into lactate with activation of pyruvate dehydrogenase kinase1 (PDK-1), lactic dehydrogenase (LDH-A) and monocarboxylate lactate transporter (MCT-1). This results in pyruvate being diverted toward lactate (6, 122). In the cytosol, pyruvate is converted into lactate through activation of lactic dehydrogenase (LDH-A). In cancers, this phenomenon is referred to as aerobic glycolysis or Warburg effect (123). PDK1 phosphorylates the pyruvate dehydrogenase complex which is inhibited and prevents the conversion of pyruvate into acetyl-CoA in mitochondria. This leads to decreases acetyl-CoA entering the tricarboxylic acid cycle. In addition, the WNT/β-catenin pathway induces the transcription of the genes Cyclin D1 and cMyc (124) (Figure 3). cMyc activates LDH-A (125) and increases the hypoxia-inducible factor-1α which controls PDK-1 (126). Conversely, PPARγ activation selectively decreases PDK mRNA (127). To date, complex cellular metabolism through canonical WNT/β-catenin and PPARγ pathways is not fully detailed in BPD.

## GSK-3β in PBD

GSK-3β (inactive state corresponding to the WNT on-state) is involved in the differentiation of mesenchymal stem cells (MSCs) into myofibroblasts (128). GSK-3β is down-regulated in mesenchymal myofibroblastic cells obtained by means of tracheal aspiration in infants with BPD (120). GSK-3β kinase activity can be inhibited by several factors such as WNTs, TGF-β, BMPs, serotonin, endothelin, cardiotrophin, and connective tissue growth factor (CTGF) (129). In addition, CTGF activates the TGF-β receptor. During lung development, hyper-expression of both TGF-β (130) and CTGF leads to a pulmonary phenotype similar to that of BPD (63). During the neonatal period, hyper-expression of CTGF induces thickening of alveolar septa and differentiation of MSCs into myofibroblasts, decreases the formation of secondary septa (63), and increases the risk of pulmonary hypertension (131). Both TGF-β and CTGF are likely to activate each other (132–134) (Figure 3). In mice lung, hyper-expression of CTGF induces phosphorylation of Ser9 GSK-3β and the translocation of β-catenin to the nucleus of type II alveolar cells (131).

## PPARγ and PBD

Stretch on alveolar type II cells induces the expression of parathyroid-hormone-related protein (PTHrP) which binds to the PTHrP receptor. PTHrP is expressed in lipofibroblasts and upregulates PPARγ via protein kinase activation. PPARγ induces lung transdifferentiation of myofibroblasts into lipofibroblasts. Moreover, lipofibroblasts stimulate the proliferation and differentiation of alveolar type II cells, thus contributing to alveolarization (113). Lipofibroblasts synthesize leptin, which leads to stimulation of the synthesis of *de novo* surfactant phospholipid. The decrease in PTHrP in alveolar type II cells, after exposure to hyperoxia, volutrauma, inflammation and infections, downregulates the alveolar lipofibroblast PPARγ expression (118, 119, 135) and induces lipofibroblast-myofibroblast transdifferentiation (97, 113, 136). PPARγ prevents these deleterious effects (97), reducing inflammation processes by inhibiting NF-κB and rosiglitazone (RGZ) by decreasing lung infiltration by neutrophils (137). PPARγ induces angiogenesis after direct stimulation of endothelial cells through angiogenic growth factors and cytokines that stimulate endothelial cells that participate in the maintenance of alveolar structures (138, 139). Moreover, the pulmonary vasculature is dramatically impaired in human premature infants dying from BPD. Thus, VEGF and VEGF receptors are diminished in their lungs (59). Disruption of the VEGF signaling stops the alveolar and pulmonary vascular development (140). This strongly suggests that PPARγ plays an important role in the microvascular development in the lungs (115, 141).

## PPARγ Agonists and BPD

Numerous studies have shown that PPARγ agonists can play a positive role in animal models of BPD. Angiogenesis is enhanced by VEGF which improves both alveolar and pulmonary vascular development in neonatal rats (142, 143). VEGF plays a major role in the microvascular development of the lungs (138). In rat exposed to intra-amniotic LPS and postnatal hyperoxia, the angiogenic growth factor is decreased, a situation that is alleviated by RGZ (144). Hyperoxia induces a decrease in VEGF and the platelet endothelial cell adhesion molecule (PECAM-1or CD31) and an increase in fibronectin. This is blocked after pioglitazone (PGZ) nebulization. PGZ nebulization also blocks modifications in lung interleukins (IL-6 and IL-1β), chemokine ligand 2 (CCL-2), pro-inflammatory cytokine MIF, the BcL2/Bax protein ratio and lung morphometry (radial alveolar count). RGZ restores alveolar and pulmonary vascular development and lessens pulmonary hypertension (145). In a similar rat model, RGZ has also been found to restore VEGF and its receptor VEGF R-2 in the lungs (144). This protective effect of RGZ may be induced via VEGF enhancing the angiogenic activity. In an animal model, the systemic administration of RGZ, either antenatally or postnatally, enhances lung maturation and can prevent hyperoxia-induced acute neonatal lung injury. This strongly suggests that PPARγ agonists could potentially play an important role in preventing and/or treating BPD (53, 115, 146, 147).

In rat pup lung, RGZ increases the expression of the PTHrP receptor and surfactant protein-B (115). The inhibitory effects of intra-amniotic LPS and postnatal hyperoxia on alveolarization are alleviated by RGZ, which induces a protective effect on it via an increased PPARγ expression in the alveolar lipofibroblasts. RGZ significantly enhances lung vascular maturation during normal lung morphogenesis in rat pups (115). In rat, after exposure to hyperoxia, the level of PPARγ protein decreases but increases after PGZ nebulization (145). PPARγ ligands inhibit fibroblast activation through TGF-β1. RGZ and ciglitazone (CGZ) inhibit profibrotic changes in TGF-β1-stimulated serum-deprived A549 cells (from the human AEC cell line), independently of the inhibition of the Smad pathway. Their inhibitory effects on changes in collagen I and E-cadherin (epithelial cell marker) appear to be PPARγ-dependent. PPARγ agonists inhibit pulmonary myofibroblast differentiation by TGF-β and collagen synthesis (148). In newborn rat, nebulized PGZ significantly improves hyperoxia-induced abnormalities via TGF-β pathway activation. PGZ-nebulization blocks Smad3 and 7, and TGF-β II receptor protein levels increased after hyperoxia exposure.

In newborn rat, the nebulized PPARγ agonists RGZ and PGZ increase the expression of alveolar epithelial (apolipoproteins SPB-SPC, surfactant phospholipid) and mesenchymal (adipose differentiation-related protein) markers of lung maturation and decrease markers of lung injury (BcL2, Bax), and markers of TGF-β activation (145). Human fibroblasts treated with PPARγ agonists blocked the TGF-β signaling. SMAD3 or SMAD4 knockdown suppresses the effects of TGF-β on PPARγ mRNA and protein expression (149). In cultured human lung fibroblasts, the increases in α-SMA expression and the fibrillary collagen content induced by TGF-β are prevented by RGZ, TGZ and CGZ (148, 150). TGF-β1 induces EMT, and epithelial cells acquire a phenotype of mesenchymal cells (89, 151, 152). RGZ and CGZ inhibit several TGF-β1-induced changes in EMT markers as well as lung fibrosis (153).

## Canonical WNT-TGF-β-PPARγ Axis in Diseases Other Than BPD

Targeting the canonical WNT/β-catenin-TGF-β-PPAR**γ** axis by upregulating PPAR**γ** and downregulating the canonical WNT/TGF-β pathways might be of interest with respect to improving the development of the alveolar stage of BPD at birth and minimizing pulmonary fibrosis later during childhood and adulthood. Numerous studies have demonstrated the potential role of PPARγ agonists and the inhibition of canonical WNT/TGF-β pathways in many pathologies leading to pulmonary fibrosis outside of BPD.

### PPARγ Agonists

PPARγ agonists are considered part of a potential future strategy that would target PPARγ activity or expression as a therapy for controlling fibrosis (154). In human lung, PPARγ agonists inhibit profibrotic phenotype fibroblasts and pulmonary fibrosis induced by bleomycin (150). In rat, PGZ improves bleomycin-induced acute lung injury and fibrosis (155). In a bleomycin-induced model of lung fibrosis, PPARγ agonists induce an anti-fibrotic activity (96, 148, 150) and RGZ attenuates fibrotic effects in rats (156). In mouse, bleomycin-induced lung injury, RGZ and 15-deoxy-Δ12,14-prostaglandin J2 significantly reduce lung abnormalities (157). In scleroderma, the skin fibrotic phenotype of fibroblasts is alleviated by RGZ (158). Bleomycin-induced scleroderma is abrogated by RGZ and this blocks profibrotic effects (159). RGZ induces an antifibrotic effect in scleroderma lung fibroblasts (160). In systemic sclerosis, PPARγ activation induces protection against an excessive fibrosis and may represent a therapeutic target (161). The PPARγ agonist PGZ inhibits fibrosis in rat liver after a choline-deficient L-amino acid defined diet (162, 163). PPARγ ligands exert notable antifibrotic effects in human lung fibroblasts (164). RGZ inhibits differentiation, migration, and proliferation in cultured human lung fibroblasts (165). In a murine model of neutrophilic asthma, RGZ diminishes airway inflammation by inhibiting T cell proliferation (166). PPARγ is expressed in airways and inhibits features of airway remodeling in a mouse asthma model (167). TZDs increase the WNT inhibitor Dickkopf-1 in adipocytes (168). In fetal rat lung exposed to LPS, RGZ exerts protective effects against inflammation through PTHrP-driven epithelial-mesenchymal interactions (169). RGZ increases angiogenesis and may represent a new potential therapy for improving lung development in extremely premature infants. In rat pups, nebulized PPARγ agonists RGZ and PGZ augment neonatal lung maturation and injury repair by increasing the expression of markers of alveolar epithelial and mesenchymal maturation via stimulation of alveolar interstitial lipofibroblast maturation and by providing protection against hyperoxia-induced lung injury (145). In lung epithelial cells in chronic obstructive pulmonary disease, down-regulated PPARγ increases the likelihood of a proinflammatory phenotype (170).

### Inhibition of TGF-β and Canonical WNT/β-catenin

In mesangial cells, TGF-β upregulates the type I collagen and this is blocked by PPARγ activation (171). In hypertrophic scar fibroblasts, induction of CTGF and extracellular matrix by TGF-β1 is inhibited by PPARγ (172). Fibrosis may be attenuated by blocking TGF-β1 by means of PPARγ agonists (173). PPARγ abrogates Smad-dependent collagen stimulation by targeting the p300 transcriptional coactivator (174). In murine lung fibroblasts, TGF-β1 controls PPARγ expression, transcription and activity through Smad3 signaling (175). Several drugs targeting the TGF-β pathway have been used in phase III clinical trials in oncology and against fibrosis and radiation lesions (176). In human hepatic stellate cells, the TGF-β1/Smad3-pathway is prevented by PPARγ (177). In systemic sclerosis, abnormal TGF-β expression is involved in fibrosis. Small-molecule inhibitors of TGF-β-receptor activity are effective in animal models of fibrosis. Imatinib mesylate and related tyrosine kinase inhibitors block TGF-β signaling and abrogate fibrotic processes (178). In systemic sclerosis, the aberrant PPARγ function is involved in fibrosis in skin and lungs. The antifibrotic effects seem to be related to the inhibition of TGF-β/Smad signal transduction (99). In fibroblasts, TGF-β downregulates PPARγ in systemic sclerosis (102). The *TGF-*β*1* gene is repressed by PPARγ through the PTEN-mediated p70 ribosomal S6 kinase-1 inhibition (179). TGF-β suppresses PPARγ expression via SMAD binding (149) (Figure 3). The canonical WNT/β-catenin pathway represents a potential therapeutic target for fibrosis (180). In systemic sclerosis, the canonical WNT/β-catenin pathway is upregulated and induces a Smad-dependent fibrotic effect in mesenchymal cells (98). Inhibition of the β-catenin pathway improves renal interstitial fibrosis (181). Activation of the canonical Wnt/β-catenin signaling inhibits the GSK3-β and induces dermal fibrosis (182).

## Hypoxia, PPARγ, and PPARγ Agonists

In very premature infants, the alveolar stage is very abnormal and the thickening of the alveolar walls makes gaseous exchanges difficult, leading to hypoxia and hypercapnia. However, chronic hypoxia itself impairs lung development (38, 39). In the newborn lung animal model, hypoxia enhances the TGF-β pathway and inhibits alveolar development, resulting in pulmonary arterial abnormalities. PPARγ can be reduced by increasing TGF-β (85, 183). Hypoxia inhibits PPARγ (40, 184), but increases TGF-β signaling, which is attenuated by RGZ. Importantly, RGZ attenuates hypoxia-induced inhibition of lung development (40). In newborn mouse lung, chronic hypoxia diminishes the levels of PPARγ mRNA expression and PPARγ protein. After hypoxia exposure the oral administration of RGZ restores PPAR-γ mRNA and protein levels (40). RGZ diminishes hypoxia-induced inhibition of alveolar development and prevents hypoxia-induced impairment of the respiratory function. PPARγ is decreased by hypoxia, a decrease that is prevented by inhibition of the TGF-β pathway.

## Deleterious Effects of PPARγ Agonists

TZDs can have adverse effects. The clinical administration of PGZ and RGZ has revealed several adverse effects, including weight gain, bone fractures, fluid retention and congestive heart failure (185–187). In the USA, TGZ was removed from the market in 2000 due to liver failure in diabetic patients (188). RGZ has been withdrawn due to hepatotoxicity which has not been observed with RGZ and PGZ (189). RGZ is associated with an increased risk of strokes, myocardial infarction, heart failure and even death (190, 191). However, it has been shown that there is no increase in cardiovascular incidents with RGZ therapy (192, 193). As for PGZ, it has been associated with bladder cancer, bone fracture and heart failure (194). Few selective PPARγ modulators have been synthesized and these have been tested for safety and therapeutic efficacy. Thus, the PPARγ ligand SR1664 has been found to improve insulin sensitivity and does not induce adverse effects with regard to fluid retention or bone formation (195). SR1664 binds PPARγ with high affinity and blocks the PPARγ-Cdk5-mediated phosphorylation. Great efforts are done to improve the safety of the first-generation of PPARγ agonists. It appears important to understand the complex PPARγ regulation by integrating the role of coactivators and corepressors and the role of posttranslational mechanisms. It would seem important to develop new tissue-specific PPARγ agonists. In the case of BPD, lung-specific nebulized PPARγ agonists could enhance the therapeutic benefits and reduce the deleterious effects.

## Conclusion

Significant prematurity predisposes to general hypoxia, particularly in the pulmonary tissue. Both hypoxia and hyperoxia promote upregulation of the canonical WNT/β-catenin system and TGF-β and downregulation of PPARγ. This stops lung maturation and gives rise to the transdifferentiation of lipofibroblasts into myofibroblasts, which may lead to pulmonary fibrosis and severe pulmonary sequelae. Since hypoxia and hyperoxia have similar consequences for the TGF-β/WNT/β-catenin/PPARγ pathways, this requires a fine tuning of oxygen use in very premature infants. Numerous studies have shown PPARγ agonists have a beneficial effect on lung maturation and may provide a potentially safe lung therapy (53, 54, 115, 146, 147, 157, 196, 197). Nebulized PPARγ agonists may represent a potential strategy for improving postnatal lung maturation, both by inhibition of the TGF-β/WNT/β-catenin pathway and by preventing neonatal hyperoxia-induced lung injury in premature BPD infants, thereby reducing morbidity and mortality.

## Author Contributions

All the authors contributed equally to the final version of the manuscript and approved the submitted version.

### Conflict of Interest Statement

The authors declare that the research was conducted in the absence of any commercial or financial relationships that could be construed as a potential conflict of interest.
